# Mango Peels as an Industrial By-Product: A Sustainable Source of Compounds with Antioxidant, Enzymatic, and Antimicrobial Activity

**DOI:** 10.3390/foods13040553

**Published:** 2024-02-11

**Authors:** Nika Kučuk, Mateja Primožič, Petra Kotnik, Željko Knez, Maja Leitgeb

**Affiliations:** 1 Faculty of Chemistry and Chemical Engineering, University of Maribor, Smetanova ulica 17, 2000 Maribor, Slovenia; nika.kucuk@um.si (N.K.); mateja.primozic@um.si (M.P.); petra.kotnik@um.si (P.K.); zeljko.knez@um.si (Ž.K.); 2 Faculty of Medicine, University of Maribor, Taborska ulica 8, 2000 Maribor, Slovenia

**Keywords:** *Mangifera indica*, peels, bioactive substances, LC-MS/MS, proteins, enzymes, antibacterial activity

## Abstract

Plant waste materials are important sources of bioactive compounds with remarkable health-promoting benefits. In particular, industrial by-products such as mango peels are sustainable sources of bioactive substances, with antioxidant, enzymatic, and antimicrobial activity. Appropriate processing is essential to obtain highly bioactive compounds for further use in generating value-added products for the food industry. The objective of the study was to investigate and compare the biological activity of compounds from fresh and dried mango peels obtained by different conventional methods and unconventional extraction methods using supercritical fluids (SFE). The highest total phenolic content (25.0 mg GAE/g DW) and the total content of eight phenolic compounds (829.92 µg/g DW) determined by LC-MS/MS were detected in dried mango peel extract obtained by the Soxhlet process (SE). SFE gave the highest content of proanthocyanidins (0.4 mg PAC/g DW). The ethanolic ultrasonic process (UAE) provided the highest antioxidant activity of the product (82.4%) using DPPH radical scavenging activity and total protein content (2.95 mg protein/g DW). Overall, the dried mango peels were richer in bioactive compounds (caffeic acid, chlorogenic acid, gallic acid, catechin, and hesperidin/neohesperidin), indicating successful preservation during air drying. Furthermore, outstanding polyphenol oxidase, superoxide dismutase (SOD), and lipase activities were detected in mango peel extracts. This is the first study in which remarkable antibacterial activities against the growth of Gram-negative bacteria (*Escherichia coli* and *Pseudomonas aeruginosa*) and Gram-positive bacteria (*Bacillus cereus* and *Staphylococcus aureus*) were evaluated by determining the microbial growth inhibition rate after 12 and 24 h incubation periods for mango peel extracts obtained by different methods. Ethanolic SE and UAE extracts from dried mango peels resulted in the lowest minimum inhibitory concentrations (MIC_90_) for all bacterial species tested. Mango peels are remarkable waste products that could contribute to the sustainable development of exceptional products with high-added value for various applications, especially as dietary supplements.

## 1. Introduction

Fruit peels account for about 15–60% of waste products in fruit processing and are usually discarded [1,2,3]. A total of around 89 million tons of food waste is produced in the European Union [4,5]. Due to their high biodegradability and fermentability, fruit peels contribute to water and soil pollution, eutrophication, global warming, the greenhouse effect, etc. However, similar to the pulp, the waste parts of the fruit produced during fruit processing are a rich source of phytonutrients and valuable biologically active compounds. By applying appropriate extraction or isolation processes (low operating temperature, no use of toxic solvents) for bioactive ingredients from the waste parts of fruits, especially from peels, it is possible to obtain products with exceptional added value, which can be used mainly in the food, cosmetic, and pharmaceutical industries [6,7].

Mango (*Mangifera indica* L.) belongs to the Anacardiaceae family. It is one of the most important and popular tropical fruits for its attractive color, excellent taste and aroma, and high nutritional, phytochemical, and medicinal value [8]. The edible part is largely industrially processed into various pulps or juices, with the inedible byproducts discarded. However, mango by-products, especially peels, represent an important source of biologically active compounds that could be used for various applications. Mango by-products account for 35–55% of the total mass. Moreover, their further use could reduce the amount of waste and negative impact on the environment [9,10]. Therefore, a new approach is urgently needed to reduce their accumulation and the resulting pollution and generate value-added products.

Mango peels contain even more specific bioactive compounds than the edible part of the fruit. They are a rich source of various polyphenolic compounds (PCs), dietary fiber, pectin, carotenoids, vitamins (ascorbic acid, tocopherol), minerals (potassium, magnesium, sodium, calcium, copper, iron, manganese, zinc, chromium, phosphorus, chlorine), and enzymes. The main PCs in mango peels are gallic acid, ellagic acid, caffeic acid, ferulic acid, rutin, catechin, quercetin, and kaempferol [11,12]. It is essential to highlight the presence of mangiferin, which belongs to the xanthone class, and is the main PCs of mango. Mangiferin has antioxidant, antidiabetic, anticancer, antimicrobial, and anti-inflammatory effects [13,14]. However, the peels have been found to contain a higher concentration of mangiferin than the pulp and seeds [15,16]. In addition, some enzymes have been shown to be present in active form in mango peels, such as superoxide dismutase (SOD), catalase, peroxidase, protease, and amylase [17]. Different studies also confirmed the good antimicrobial potential of samples obtained from different parts of the mango plant [18,19]. Ethanolic extracts from the peels of different varieties of mango show an excellent inhibitory effect on the growth of various types of Gram-negative bacteria, such as *Escherichia coli*, *Salmonella typhimurium*, and *Vibrio parahaemolyticus*, and Gram-positive bacteria, such as *Staphylococcus aureus* and *Bacillus cereus* [20]. In addition, acetonic, ethanolic, and aqueous extracts obtained by the conventional maceration method exhibit efficient antimicrobial activities against various foodborne pathogenic microorganisms [21].

It is important to emphasize that the isolation of bioactive compounds is mainly influenced by the extraction method used, the operating conditions, and the extraction solvent, as different polarities of the solvents directly affect the extraction efficiency [22]. However, the quality and final composition of the extract obtained are mainly influenced by the method of cultivation and location of the plant, weather conditions, plant species, stage of development, the process and temperature of drying and storage, and the final water content of the raw material [23]. Moreover, the antimicrobial activity of the extracted samples is related to the content and synergistic effects of the bioactive substances in the extracts obtained [24].

Some sensitive but important bioactive substances may be degraded during drying and storage. On the other hand, harmful microorganisms may develop if the fresh peels are not processed immediately and when unsuitable drying conditions are used [25,26]. Therefore, the present study focused on the determination of the bioactive compound content in fresh and dried mango peels obtained by different extraction methods. Dried mango peels were obtained by air-drying at room temperature, as this is considered one of the simplest, least demanding, and most economical methods [27]. On the other hand, excessively high temperatures during oven-drying can degrade bioactive compounds [28]. While oven-drying is energy inefficient, it is a cost-effective method [29]. Sun-drying is also a cost-effective method, but it is more susceptible to microbial contamination due to unhygienic conditions. It can also have a negative effect on the plant material by reducing the nutrient content and causing color changes due to direct exposure to UV radiation [30,31]. Although freeze-drying is one of the most suitable methods to dry plant material and successfully preserve valuable substances, it requires very high investment and operating costs, which is very unfavorable [32,33].

Furthermore, the study compares conventional methods (Soxhlet method (SE), ultrasound-assisted method (UAE), homogenization-assisted method (HAE)) and an unconventional technique (supercritical fluid technique (SFE)) to obtain the bioactive compounds from mango peels. The main objective of the study was to investigate the content of bioactive compounds in terms of total phenols (TPC), proanthocyanidins (PACs), certain important PCs, and antioxidant activities. In addition, the total protein (TP) content and activities of specific enzymes in mango peel extracts were determined for the first time. Furthermore, one of the main objectives was also to validate the antimicrobial activity against various Gram-negative and Gram-positive bacterial strains and fungi. To the best of our knowledge, this is the first study to quantitatively evaluate the growth inhibitory properties of mango peel extracts against bacteria, determined as microbial growth inhibition rates (MGIRs).

This study makes a significant contribution to raising awareness of the increasing use of underutilized mango by-products, which, due to their high content of important bioactive compounds, contribute to the production of high-value-added products that can be used in various industries (food, cosmetics, biomedicine, pharmaceuticals), focusing on the use of mango peels dried by a low-cost air-drying process.

## 2. Materials and Methods

### 2.1. Chemicals and Reagents

The following chemicals were used in the study: malt extract, potato dextrose broth, Triton X-100, and tryptic soy broth, obtained from Fluka, Buchs, Switzerland. Mueller–Hinton broth (MHB) and potato dextrose agar (PDA) were purchased from Biolife, Milano, Italy. In addition, 4-Aminoantipyrine (4-APP), Coomassie Blue G-250, ethanol (EtOH, ≥99.5%), hydrochloric acid (HCl, 37.0%), meat extract, meat peptone, phosphoric acid (85.0%), potassium dihydrogen phosphate (KH_2_PO_4_), sodium chloride (NaCl), sodium dihydrogen phosphate monohydrate (NaH_2_PO_4_·H_2_O), and sodium hydrogen phosphate (Na_2_HPO_4_) were obtained from Merck, Darmstadt, Germany. Calcium chloride (CaCl_2_), D-(+)-glucose anhydrous, and ferrous sulfate heptahydrate (Fe(SO_4_)·7H_2_O) were purchased from Kemika, Zagreb, Croatia. Carbobenzoxy-L-Glutaminylglycine (CBZ-Gln-Gly) was obtained from Zedira GmbH, Darmstadt, Germany. Acetic acid (glacial, ≥99.7%), agar, 2,2′-azino-bis(3-ethylbenzothiazoline-6-sulfonic) acid, bovine serum albumin (BSA), caffeic acid (99.0%), casein, (+)-catechin (≥98.0%), chlorogenic acid (≥99.0%), L-3,4-dihydroxyphenylalanine (L-DOPA, ≥98.0%), 3,5-dinitrosalicylic acid (DNS), 2,2-diphenyl-1-picrylhydrazyl (DPPH, ≥97.0%), ellagic acid (≥95.0%), ethylenediaminetetraacetic acid (EDTA, 98.5–101.5%), Folin–Ciocalteu’s phenol reagent, gallic acid (GA, ≥97.5%), glucose assay, hesperidin/neohesperidin (≥97.0%), hydrogen peroxide (H_2_O_2_), hydroxylamine hydrochloride (HONH_2_·HCl), L-glutamic acid (99.0%), L-glutathione reduced (≥98.0%), maltose, mangiferin (≥98.0%), methanol (MeOH, ≥99.9%), yeast extract, peptone from soybean, p-nitrophenyl butyrate (p-NPB, ≥98.0%), potassium sodium tartrate tetrahydrate (KNaC_4_H_4_O_6_·4H_2_O, 99.0%), pyrogallol (≥99.0%), Sigmacell cellulose, sodium acetate (CH_3_COONa, ≥99.0%), sodium carbonate (Na₂CO_3_, ≥99.5%), sodium hydroxide (NaOH, ≥95.0%), starch, trichloroacetic acid (TCA, ≥99.0%), rutin (≥97.0%), and Trizma Base (NH_2_C(CH_2_OH)_3_, ≥99.7%) were purchased from Sigma-Aldrich, St. Louis, MO, USA. Carbon dioxide (CO_2_, purity 2.5) was obtained from Messer, Ruše, Slovenia. Ciprofloxacin (400 mg/200 mL) was obtained from University Medical Centre Maribor.

### 2.2. Microorganisms

Selected bacteria (*B. cereus* (DSM 345), *E. coli* (DSM 498), *Pseudomonas aeruginosa* (DSM 1128), *S. aureus* (DSM 346)) and fungi (black mold *Aspergillus brasiliensis* (DSM 1988), yeast *Candida albicans* (DSM 1386)) were purchased from DSMZ-German Collection of Microorganisms and Cell Cultures GmbH from Berlin, Germany.

### 2.3. Preparation of Plant Material

Washed, fully ripe, Keitt-type mango fruits (country of origin: Puerto Rico) were peeled with a fruit peeler. Some of the obtained peels were air-dried without pretreatment at room temperature and protected from direct sunlight to avoid possible degradation of bioactive compounds. Before the extraction process, the completely dried peels were ground to a uniform size. The mean particle size was determined by sieve analysis and amounted to 1.0 mm. The dried peels were then stored at room temperature until further use. The remainder of the fresh peels was used immediately to avoid possible microbial contamination and putrefaction processes. The fresh peels were cut into smaller pieces and then subjected to an extraction process.

### 2.4. Moisture Content

The moisture content in fresh and dried mango peels was analyzed with the Mettler Toledo HX204 Halogen Moisture Analyzer. The moisture content was determined according to the thermogravimetric principle based on the weight loss of the dried analysis material by heating to 115 °C.

### 2.5. Thermogravimetric Analysis/Differential Scanning Calorimetry

The mango peels (fresh and dried) were subjected to thermal stability testing, including thermogravimetric analysis/differential scanning calorimetry (TGA/DSC). Both analyses were performed simultaneously on a TGA/DSC instrument (TGA/DSC1, Mettler Toledo AG (MTANA), Zurich, Switzerland) in a nitrogen atmosphere. Samples were weighed into aluminum pans and analyzed at a temperature range from 25 to 600 °C and a rate of 10 °C/min.

### 2.6. Extraction Procedures

Three different conventional extraction methods, namely SE, UAE, and HAE, were used to extract bioactive compounds from fresh and dried mango peels. Two polar extraction solvents, H_2_O and EtOH, were used. In addition, unconventional SFE using supercritical carbon dioxide (SC CO_2_) and EtOH as a co-solvent was also performed. For this method, the use of dry raw material is mandatory. Therefore, only the extraction of dried mango peels was performed. All extraction solvents used are considered GRAS (Generally Recognized as Safe).

#### 2.6.1. Soxhlet Extraction

SE was performed with both fresh and dried mango peels. Peels were transferred into a cellulose thimble and placed in a Soxhlet extraction chamber. The extraction solvent EtOH was added to a round-bottom flask connected to a Soxhlet apparatus with a reflux condenser. The solvent was then heated to reflux. The extraction was carried out until the condensate became colorless (3 refluxes). The ratio of solvent (EtOH) to fresh and dried mango peels was 5:1 and 10:1, respectively.

#### 2.6.2. Ultrasound-Assisted Extraction

UAE was performed on fresh and dried mango peels using EtOH and H_2_O as extraction solvents, respectively. Fresh or dried mango peels were mixed with an extraction solvent and sonicated at 20 °C and 40 kHz in an ultrasonic bath. Subsequently, filtering was performed through a filter flask and a Buechner funnel to remove solid, insoluble particles. The ratio of solvent (EtOH and H_2_O) to fresh and dried mango peels was 5:1 and 10:1, respectively.

#### 2.6.3. Homogenization-Assisted Extraction

HAE, using H_2_O as an extraction solvent, was performed with fresh and dried mango peels. The homogenization process was carried out for 30 min. Then, the supernatant was centrifuged to achieve complete separation of the supernatant from the insoluble material. The ratio of solvent (H_2_O) to fresh and dried mango peels was 5:1 and 10:1, respectively.

#### 2.6.4. Supercritical Fluid Extraction

SFE was performed in a semi-continuous apparatus using SC CO_2_ to obtain bioactive compounds from dried mango peels. Dried peels were placed in the autoclave and preheated to an operating temperature of 40 °C in a water bath. The CO_2_ was compressed to an operating pressure of 200 bar and continuously introduced into the autoclave at a 2–3 mL/min flow rate. At the same time, EtOH was added as a co-solvent to achieve better extraction of the polar bioactive compounds. The ratio of the final consumption of the solvent (EtOH and CO_2_) to the dried mango peels was 5:1 and 30:1, respectively.

After each extraction procedure, the extraction solvent (EtOH or H_2_O) was evaporated using a rotary evaporator (Büchi^®^ Rotavapor R-144, Flawil, Switzerland). The extracts were stored in a freezer at −20 °C until further analysis.

#### 2.6.5. Extraction Yield Determination

Each individual extraction was performed three times. The extraction yield was calculated as the ratio between the weight of the extract obtained and the initial weight of the material used. The results are given as mean values.

### 2.7. Quantitative Determination of Total Phenols and Proanthocyanidins

#### 2.7.1. Determination of Total Phenolic Content (TPC)

The TPC content in the extracts obtained was determined by the Folin–Ciocalteu method. The analysis procedure for TPC is described in detail by Škerget et al. [34]. The results are given as mean values and are expressed as mg of GA equivalents (GAE) per gram of fresh weight (FW) for fresh peels or dry weight (DW) for dried peels.

#### 2.7.2. Determination of Total Proanthocyanidin Content (PAC)

Condensed tannins or PAC were determined by a colorimetric method using HCl and *n*-butanol, in which acid-catalyzed oxidative depolymerization of PAC leads to the formation of the corresponding anthocyanidin compounds [34]. The results are reported as mean values expressed as mg of PAC per g of FW or DW.

### 2.8. Determination of Antioxidant Activity

The antioxidant activity of the extracts was determined using the stable free radical 2,2-diphenyl-1-picrylhydrazyl (DPPH) [35]. Antioxidant activity was expressed as a percentage of inhibition regarding the reference solution.

### 2.9. Liquid Chromatography with Tandem Mass Spectrometry Analysis

Liquid chromatography with mass spectrometry (LC-MS/MS) was used for the identification and quantitative determination of selected phenolic compounds in mango peel extracts, using Agilent 1200 HPLC together with Agilent 6464 QQQ with JetStream technology, as described in detail by Hrnčič et al. [36].

### 2.10. Determination of Total Protein (TP) Content 

The Bradford method [37] was used to determine TP content using bovine serum albumin (BSA) as the standard. All analyses were performed in three replicates and expressed as mean values in mg of protein per g of FW or DW.

### 2.11. Determination of Enzyme Activities

The enzyme activity of α-amylase [38], cellulase [39], glucoamylase [40], laccase [41], lipase [38], peroxidase [35], polyphenol oxidase (PPO) [42], protease [39], SOD [43], and transglutaminase (TGM) [44] were examined with specific spectrophotometric enzymatic assays for each selected enzyme using a UV spectrophotometer (Varian—CARY^®^ 50 UV–VIS Spectrophotometer, Varian Inc., Middelburg, The Netherlands). Each assay was performed in three replicates. The results are reported as mean values expressed in units (U) per g of protein.

### 2.12. Determination of Antimicrobial Activity

For the determination of the inhibitory properties of the extracts obtained on the growth of selected microbial cells (Gram-negative bacteria (*E. coli* and *P. aeruginosa*), Gram-positive bacteria (*B. cereus* and *S. aureus*), and fungi (black mold *A. brasiliensis* and yeast *C. albicans*)), the qualitative disk diffusion method and the quantitative microbroth dilution method were used.

#### 2.12.1. Qualitative Disk Diffusion Method

The antimicrobial properties of the mango peel extracts were verified by the qualitative Kirby–Bauer disk diffusion method, which was previously described in detail in the study by Kupnik et al. [45], using 6 mm sterile cellulose discs. Ciprofloxacin was used as a positive control. All tests were performed with three replicates, and the results are given as mean values.

#### 2.12.2. Quantitative Microbroth Dilution Method

The microbroth dilution method was used, which is best suited for the in vitro determination of the susceptibility or resistance of microorganisms [45]. Therefore, the minimum inhibitory concentrations (MICs) and the MGIRs were determined. The method was performed only for those microorganisms that were found to be susceptible when exposed to the mango samples, as determined by the disk diffusion method. All experiments were performed in three replicates and are expressed as mean values.

### 2.13. Statistical Analysis

All statistical data analyses were performed using IBM^®^ SPSS^®^ Statistics. Statistical data analysis was performed to study the differences between extraction methods. The normality of the distribution of data was tested using Shapiro–Wilk’s test. The homogeneity of variances was determined using Levene’s test. Differences between extractions were determined with one-way analysis of variance (ANOVA) followed by Tukey’s post hoc test (for normally distributed data) and with the nonparametric Kruskal–Wallis H test, followed by pairwise comparison using the Dunn–Bonferroni post hoc method (for abnormally distributed data).

## 3. Results and Discussion

The present study focused on the influence of various parameters (drying method, extraction method) on obtaining highly active bioactive compounds with good antimicrobial and antioxidant activity.

### 3.1. Thermal Stability of Mango Peels

For comparison, the moisture content in fresh and dried mango peels was first analyzed. Fresh mango peels contained 77.74% moisture, while completely air-dried peels stored at room temperature in an airtight container still contained 11.06% moisture.

**Figure 1:**
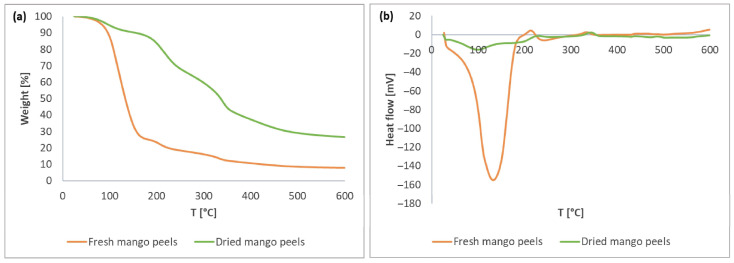
TGA (**a**) and DSC (**b**) of fresh and air-dried mango peels.

The differences in thermal behavior of fresh and dried mango peels were further investigated using TGA/DSC analysis. Figure 1a shows the thermal degradation of fresh and dried mango peels as determined by thermogravimetric analysis. Due to the high moisture content, fresh mango peels showed a drastic weight loss in the temperature range of 39–202 °C. On the other hand, the weight of dried mango peels gradually decreased throughout the entire temperature range, decreasing by 73.3%. The total weight loss of fresh peels was 92.0%. The two curves are generally comparable after excessive evaporation of moisture in the fresh peels, indicating a similar composition. The residue represents the ash fraction, which contains minerals that cannot be degraded [46]. The residue accounted for 8.0% of the fresh peels and 26.7% of the dried peels, based on the total mass of the studied peel samples.

Figure 1b shows the DSC thermogram for fresh and dried mango peels. An initial melting peak at 99 °C for the dried peels and at 133 °C for the fresh mango peels indicates the evaporation of H_2_O and the beginning of the degradation of the carbohydrates, indicating the melting point (endothermic peak). For example, the melting point of the different sugars is 126.5 °C for fructose, 159.6 °C for glucose, and 190.4 °C for sucrose [47], which are present in mango fruit [48]. A slight shift in the melting peak in fresh peels is due to the different binding of H_2_O and sugars compared to the dried peels. Moreover, the two curves of fresh and dried mango peels do not differ further. The second peak (198–254 °C) and the third peak (304–368 °C), shown as exothermic peaks, are due to the degradation of long-chain, high-molecular-weight polymers [46] or various salts, for example, sulfur-containing salts, such as sodium hydrogen sulfite. Sodium hydrogen sulfite, which decomposes at 315 °C, is classified as a food additive [49]. Therefore, it can be applied to the surface of mango peels during post-harvest treatment to preserve their freshness and prevent them from rotting too quickly and experiencing discoloration during long transportation periods.

Since the thermal stability of fresh and dried peels was comparable, we performed further extractions of differently dried peels to compare bioactive compound content and biological activity.

**Figure 2:**
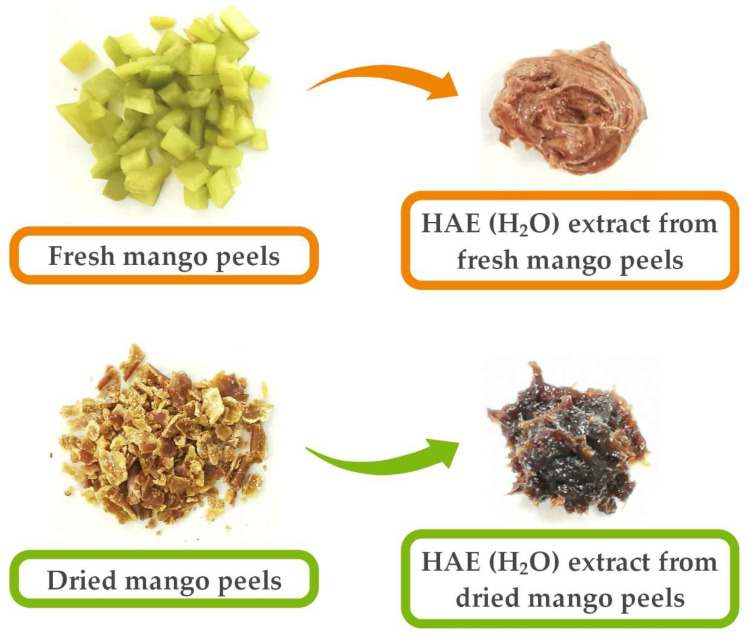
Plant material used (fresh and dried mango peels) and HAE (H_2_O) extracts obtained from fresh and dried mango peels.

Furthermore, when visually assessing the color of the plant material used (fresh or dried peels), it was found that the dried peels had a darker color due to the lower moisture content. Consequently, the extracts obtained also had a slightly darker color (Figure 2). Therefore, the color of the material may also be related to a change in the content of bioactive compounds.

### 3.2. Extraction Yield Determination

**Figure 3:**
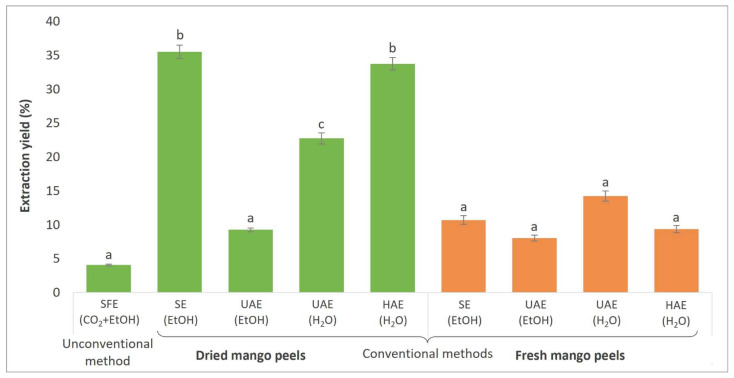
Extraction yields obtained by different extraction procedures (SFE—supercritical fluid extraction; SE—Soxhlet extraction; UAE—ultrasound-assisted extraction; HAE—homogenization-assisted extraction) from fresh or dried mango peels with different solvents (CO_2_ + EtOH, EtOH, H_2_O). Different letters indicate significant difference (*p* < 0.05).

Different extraction methods were performed using fresh or dried mango peels as raw material to compare the effects of the extraction procedure (conventional and unconventional) and the extraction solvent (CO_2_ + EtOH, EtOH, H_2_O) used on the profile of bioactive compounds and biological activity. Figure 3 presents the efficiency of each extraction performed, expressed as extraction yield (%).

The highest extraction yield was obtained with conventional extraction techniques (SE (EtOH) from dried peels (35.52%) and HAE (H_2_O) from dried peels (33.76%)). The explanation lies in the prolonged action of the extraction solvent (EtOH) on the mango peels using SE. During HAE, efficient cell wall disruption was achieved, causing the polar bioactive compounds of the cell wall to dissolve in H_2_O. However, the operating temperature in SE was above the boiling temperature of EtOH (>78 °C), which could have a negative effect on the extraction yield of sensitive bioactive compounds. In the other extraction techniques performed, the operating temperature was lower (40 °C in the case of SCF, and 22 °C for HAE and UAE).

On the other hand, when comparing UAE with the different extraction solvents used, higher extraction yields were obtained with H_2_O as the extraction solvent than with EtOH, which can be attributed to the greater polarity of H_2_O. In general, a higher amount of extract was obtained from the dried peels. This is due to the large amount of moisture in fresh peels. Dorta et al. [50] also obtained higher extraction yields when fresh peels were extracted by microwave-assisted extraction (MAE) compared to dried peels by different drying methods. The extraction yields of air-dried mango peels determined in a study by Souza et al. [51] are in line with our results.

Regarding SFE, since CO_2_ is a nonpolar solvent, a polar co-solvent such as EtOH should be used to allow the extraction of important polar bioactive compounds. Therefore, the efficiency of SFE was the lowest compared to conventional methods. Sánchez-Camargo et al. [52] optimized the SFE operating conditions for mango peel extraction, with extraction yields ranging from 1.60 to 6.25%. The highest yields were obtained at operating conditions of 300 bar and 60 °C and using EtOH as a co-solvent. This confirms the presence of polar secondary metabolites in the plant material.

A comprehensive comparison with the results of other studies is difficult because the operating conditions of the extractions performed and the extraction solvent used are different. This significantly affects both the extraction yield and the phytochemical and nutraceutical profile of the extracts. Also, usually, the origin, maturity, and growth conditions of the fruit are not the same.

### 3.3. Content of Secondary Metabolites in Mango Peel Extracts and Their Antioxidant Activity

**Figure 4:**
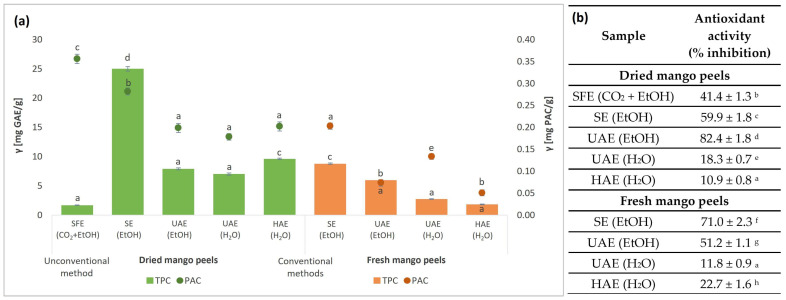
Content of total phenols (TPC) and proanthocyanidins (PAC) (**a**) and antioxidant activity (**b**) of mango peel extracts from fresh and dried peels obtained by different extraction procedures (SFE—supercritical fluid extraction; SE—Soxhlet extraction; UAE—ultrasound-assisted extraction; HAE—homogenization-assisted extraction) with different solvents (CO_2_ + EtOH, EtOH, H_2_O). Different letters indicate significant difference (*p* < 0.05).

A quantitative investigation of the content of TPC and PAC of the extracts obtained from fresh and dried mango waste was performed. The results are shown in Figure 4a.

The highest TPC content was achieved in samples obtained by SE (EtOH) (25.0 mg GAE/g DW), as seen in Figure 4a. Since the lowest extraction yield was obtained in the SFE, consequently, the TPC content was also the lowest (1.7 mg GAE/g DW). The extracts obtained from dried mango waste generally had higher TPC content than those obtained from fresh peels using the same extraction procedure. Furthermore, this indicates that the TPC content is maintained regardless of the air drying of the mango peels. Ethanolic extracts, also when SFE (CO_2_ + EtOH) was used, resulted in higher TPC content compared to aqueous extracts.

The content of extracted TPC during UAE is strongly influenced by operating conditions such as the solid–liquid ratio, temperature, amplitude, and time [53,54,55,56]. Borrás-Enríquez et al. [57] obtained an even higher TPC content (18.14 mg GAE/g DW) using an aqueous solution of EtOH (50%, *v*/*v*), as in our study, where separate solvents (EtOH and H_2_O) were used. The use of EtOH as an extraction solvent proved to be more efficient than the use of MeOH in the extraction of TPC [55]. Moreover, using SFE under the same operating conditions as in the present study, only without a co-solvent, a 2.4-fold lower TPC value was obtained compared to our results. This indicates that EtOH as a co-solvent significantly affects the recovery of bioactive compounds from mango by-products. Overall, the TPC values showed a tendency to decrease with increasing operating temperature and increase with increasing operating pressure [51,58].

Furthermore, the PAC content was determined in the extracts obtained from mango waste peels. PACs are colorless flavonoids that have beneficial effects on human health due to their antioxidant, anticancer, antimicrobial, anti-inflammatory, antidiabetic, neuroprotective, and hypolipidemic properties [59,60].

The results for the PAC content between ethanolic and aqueous extracts varied between 0.2 and 0.4 mg PAC/g DW and between 0.1 and 0.2 mg PAC/g FW. The highest amount of PACs was determined for the SFE extract (0.4 mg PAC/g DW), followed by the SE (EtOH) extract with 0.3 mg PAC/g DW. Thus, the operating conditions used for SFE extraction (temperature, time, pressure, addition of co-solvent) were beneficial.

According to the reviewed literature, different methods can be used to determine the PAC concentration in extracts. However, very few studies report the analysis of the content of PACs in mango peel extracts. The presence of PACs in mango peels is mainly influenced by the geographical area of the species and the extraction process [61]. The content of PACs, expressed as leucoanthocyanidin equivalents (LE), ranged from 1.2 to 5.8 mg LE/g DW, which was mainly influenced by the solvent used, while temperature had a negligible effect [62]. The highest PAC values were obtained with the mango peel extract using MeOH, EtOH:H_2_O, or Ace:H_2_O as a solvent. In another study by Dorta et al. [63], MAE yielded 0.0018–0.0048 mg LE/g DW of mango peels. The PAC content in mango peels increases with increasing fruit ripeness in different varieties. However, mango peels contain higher PAC concentrations compared to pulp, as confirmed by different studies [61,64].

The antioxidant activity of the mango peel extracts was determined using the DPPH method. This method is commonly used to evaluate the ability of substances that act as free radical scavengers. These substances are antioxidants that can donate a proton in the presence of the DPPH free radical, which is then converted into a reduced form [65]. The results were expressed as a percentage of inhibition of free radical DDPH and are presented in Figure 4b.

Furthermore, due to the different extraction methods and solvents used, different profiles of the extracted bioactive compounds were obtained. The extracts in which EtOH was used as an extraction solvent (UAE and SE) or as a co-solvent (SFE) exhibited significantly higher DPPH radical scavenging activity. This indicates that EtOH has an important influence on the extraction of bioactive compounds with antioxidant properties. The highest antioxidant activity was determined in UAE (EtOH) extract from dried peels, with 82.4% inhibition, which is 3.6 times higher than in HAE (H_2_O) extract from fresh peels (22.7%), where the highest % inhibition was achieved among aqueous extracts. On the other hand, no significant differences in the antioxidant potential of fresh and dried raw peels processed by the same method and under the same conditions were detected.

The antioxidant activity of extracts from dried mango peels obtained by UAE reached 87% DPPH inhibition using 50% (*v*/*v*) EtOH [57], which is comparable with our results obtained by UAE with 100% EtOH. Moreover, Kaur et al. [56] demonstrated the influence of operating conditions (liquid/solid ratio, temperature, amplitude, time) in the UAE on the determined antioxidant activity of mango peel extracts.

Souza et al. [51] achieved only 25% DPPH inhibition of mango peel extract obtained by SFE without a co-solvent. The present study achieved a 1.7-fold higher inhibition percentage, indicating that the co-solvent EtOH significantly improves the recovery of polar bioactive compounds with antioxidant potential when similar operation conditions are used.

### 3.4. Content of Important Phenolic Compounds in Mango Peel Extracts

Fruits are an essential source of various PCs with health-promoting properties. Therefore, a quantitative study was performed for eight important PCs, including xanthone (mangiferin), four phenolic acids (caffeic acid, chlorogenic acid, ellagic acid, gallic acid), and three flavonoids (catechin, hesperidin/neohesperidin, rutin) in the obtained mango peel extracts. The results are presented in Table 1.

Numerous studies in the reviewed literature have already shown that all parts of the mango tree, including peels, are a rich source of important bioactive compounds [66]. However, to the best of our knowledge, no study has addressed the effects of the drying process of the starting material on the retention of important bioactive compounds and their contribution to the antimicrobial activity of mango peel extracts obtained by different conventional and unconventional methods.

The results revealed that the obtained mango peel extracts varied in the composition of the analyzed bioactive compounds, considering the extraction procedure, the extraction solvent, and the raw material used (fresh or dried peels).

Mangiferin is one of the most important PCs of the mango tree having health-promoting properties [67]. Most of the analyzed extracts were found to contain mangiferin, with the highest content determined in the aqueous extracts of dried mango peels. In contrast, mangiferin was either not detected in the aqueous extracts of fresh mango peels (UAE extract) or only found in small amounts (HAE extract). The opposite results were obtained with ethanolic extracts, where a higher content of mangiferin was successfully extracted from fresh mango peels compared to dried peels. It can be concluded that air-drying has no significant effect on the possible degradation of mangiferin. Kaur et al. [68] reported that shade-drying at room temperature resulted in higher mangiferin content in the plant material than oven- or sun-drying. On the other hand, the final content of mangiferin is mainly influenced by the choice of extraction method [69] and extraction solvent [70]. In the reviewed literature, the mangiferin content in extracts obtained by different methods and operating conditions ranged from 0.331 to 150 µg/mg DW [57,67,71,72,73]. Furthermore, Marcillo-Parra et al. [74] found that the mangiferin content in mango peel extracts also varied widely depending on the mango variety, ranging from 57.2 to 3140 µg/g DW. Mangiferin is only slightly soluble in EtOH and H_2_O [75] but has a very low solubility in SC CO_2_ [76], which is consistent with our results, as the lowest mangiferin concentration was obtained with SFE (0.15 µg/g DW).

Regarding the content of phenolic acids in the extracts of mango waste peels, ellagic acid and gallic acid are the most abundant. The highest composition of ellagic acid was found in UAE (H_2_O) extract (539.42 µg/g FW) and SE (EtOH) extract (531.98 µg/g FW), while the lowest composition was present in SFE (CO_2_ + EtOH) extract (37.50 µg/g DW), as ellagic acid is poorly soluble in SC CO_2_ [77]. Furthermore, all extracts obtained from fresh mango peels had higher ellagic acid content than those obtained from dried peels by the same extraction method and under the same conditions. This indicates that the drying process may have a destructive effect on ellagic acid and its composition [78]. Ellagic acid content has already been determined in mango peel extracts, where its content ranged from 15.995 to 298.69 µg/g DW [73,79,80]. On the contrary, the highest content of gallic acid was determined in UAE (H_2_O) extract (405.72 µg/g DW), while the lowest content was found in UAE (H_2_O) extract (8.03 µg/g DW). Gallic acid is soluble in EtOH and H_2_O [81], whereas in SFE, the addition of a polar co-solvent (EtOH) is needed due to its low solubility in SC CO_2_ [82]. Extracts from dried peels were found to have a higher gallic acid composition than their fresh counterparts. Drying methods generally do not significantly affect gallic acid and its content in dried samples [83]. Higher gallic acid contents in extracts from dried peels indicate the high moisture content of fresh mango peels used for extraction. In the reviewed literature, different compositions of gallic acid in mango peel extracts have been reported, ranging from 23 to 23,816 µg/g DW [80,84,85,86,87] and 14.5 to 791.5 µg/g FW [88]. A similar phenomenon was observed in the composition of caffeic acid and chlorogenic acid. The content of the aforementioned phenolic acids was higher in extracts from dried peels than in extracts from fresh peels, comparing the same extraction procedure. This indicates that air-drying has no significant effect on caffeic and chlorogenic acids [89,90]. The highest content of chlorogenic acid was determined in SE (EtOH) extract (26.54 µg/g DW), whereas no chlorogenic acid or a very low amount was present in H_2_O extracts. Furthermore, the extract with the highest caffeic acid composition was the SE (EtOH) extract (6.60 µg/g DW), while no caffeic acid was detected in the UAE (H_2_O) extract, obtained from fresh peels. This indicates higher chlorogenic and caffeic acid solubility in EtOH than in H_2_O [91,92]. Compared with the reviewed literature, the content of chlorogenic acid in various mango peel extracts was found to be 760–2280 µg/g DW [93] and 44.05–271.9 µg/g FW [88], while the content of caffeic acid was 16.6–67.3 µg/g DW [93] and 33.03–144.3 µg/g FW [88].

Among the flavonoids, catechin was the most abundant in mango peel extracts. The highest content was observed in the SE (EtOH) extract (109.39 µg/g DW), while the lowest catechin concentration was found in the UAE (H_2_O) extract, with only 0.38 µg/g FW, since catechin is soluble in H_2_O and organic solvents, including EtOH [94]. However, in SFE, the solubility of catechin is mainly influenced by the operating conditions and the addition of a co-solvent, such as EtOH [95]. Other studies have also confirmed the presence of catechin in various mango peels, ranging from 45.73 to 115.5 µg/g DW [55,73]. A similar pattern was observed for hesperidin/neohesperidin content, except for the UAE (EtOH) extract, where more hesperidin/neohesperidin was extracted from fresh mango peels. Overall, hesperidin/neohesperidin was found in lower amounts in all extracts. The highest level was observed in the UAE (EtOH) extract (2.67 µg/g FW). Due to its polarity, hesperidin is very poorly soluble in SC CO_2_ [96]. According to the reviewed literature, to the best of our knowledge, the content of hesperidin/neohesperidin has not been determined or detected in mango peels. However, in a study by Abdel-Aty et al. [97], hesperidin was found to be the major PC in mango seed kernel extract (55.6% of total compounds). Rutin was only detected in the UAE (H_2_O) extract at a concentration of 0.80 µg/g DW. Other studies have already established that the rutin content in mango peel extracts ranges from 37.15 to 390 µg/g DW, which is mainly influenced by the mango variety [55,80].

The total content of the eight phenolic compounds analyzed was determined in the SE (EtOH) extract (829.92 µg/g DW). Overall, extracts from dried mango peels contained higher total levels of analyzed PCs than extracts from fresh peels obtained by the same extraction procedure and under the same conditions. This is an important fact, because most of the PCs analyzed were retained during the air-drying process.

### 3.5. Total Protein Content and Activities of Certain Enzymes in Mango Peel Extracts

**Figure 5:**
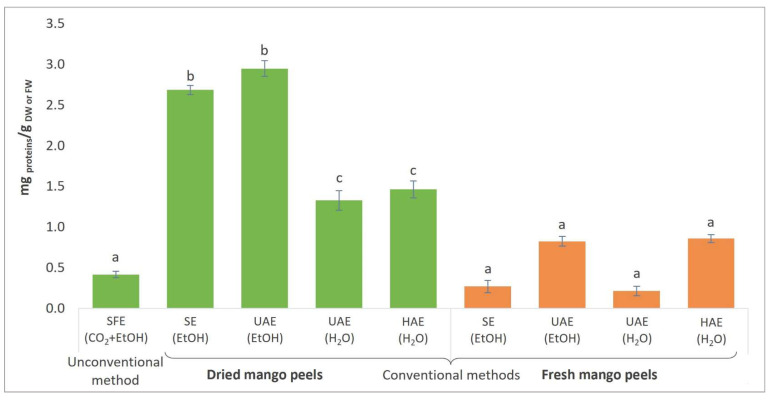
Total protein (TP) content in mango peel extracts from fresh and dried peels obtained by different extraction procedures (SFE—supercritical fluid extraction; SE—Soxhlet extraction; UAE—ultrasound-assisted extraction; HAE—homogenization-assisted extraction) with different solvents (CO_2_ + EtOH, EtOH, H_2_O). TP is expressed as mg protein per g of DW for dried peels or FW for fresh peels. Different letters indicate significant difference (*p* < 0.05).

The results of the determination of total proteins in extracts from fresh and dried mango peels are presented in Figure 5.

The highest TP content was observed in the extract obtained from dried peels using UAE (EtOH) (2.95 mg protein/g DW), as presented in Figure 5. The explanation lies in the effective mechanical disintegration of the cell wall of mango peels, causing the release of intracellular proteins. Consequently, since fresh mango peels had a high moisture content (77.74%), a higher protein concentration was observed in extracts from dried peels, indicating successful protein retention during the air-drying process. The lowest TP content was detected in the UAE (H_2_O) extract, with only 0.21 mg protein/g FW, and the SE (EtOH) extract, with 0.27 mg protein/g FW.

The TP content of fresh mango peels was 1.9 to 2.3%, based on proximate composition analysis [17,98]. Using the Bradford method, the TP content of fresh mango peels processed with acid-washed sand and sodium phosphate buffer using a mortar and pestle was estimated to be 4.0–12.7 mg/g DW. Umbreen et al. [99] determined a TP content of 6.55% in hot air-dried mango peel powder at 50–60 °C. Hot-air-dried raw and ripe mango peel powder yielded 0.16 and 0.18 mg protein/g DW, respectively [100].

However, a comparison with the total protein concentration of extracts from mango peels could not be performed because, to the best of our knowledge, no study has been published using the same extraction procedures as in the present study (SFE, SE, UAE, HAE).

Additionally, the activities of certain enzymes in mango peel extracts have been determined using specific enzymatic assays, as mango peels have been reported to contain various types of enzymes [101]. However, only a few studies are available regarding the enzymatic activities of mango peel extracts. Our main objective was to extract and identify valuable enzymes from mango peels, which could make an important contribution as an exceptional source of enzymes for further applications in various areas of medicine, cosmetics, and nutrition. The determined enzyme activities, defined as U/g protein, are shown in Table 2.

Antioxidant enzymes successfully protect fruits from oxidative stress and reduce the content of reactive oxygen compounds (ROS), as they are involved in the catalytic conversion of ROS and their by-products into stable, non-toxic molecules [102,103]. In the present study, the activities of peroxidase, PPO, and SOD were determined among the antioxidant enzymes. The activity of peroxidase was the lowest compared to the activities of all the enzymes studied. However, in the reviewed literature, peroxidase activity was significantly higher [17,98], though different substrates and methods for activity determination were used. On the contrary, the activity of PPO, responsible for enzymatic browning and melanogenesis [104], was very high in all mango peel extracts. HAE was the most outstanding technique to obtain PPO in a highly active form. The cell wall was successfully disrupted, but the mechanical force did not affect the enzyme. The PPO activities ranged from 14,036.01 to 430,837.99 U/g protein, which is higher than in a study by Tokas et al. [17] (25,800–75,500 U/g protein), where the mango peel extracts were obtained by the maceration of fresh peels. The obtained extracts from mango peels are rich in SOD, since the SOD activity was remarkable in the UAE (EtOH) extracts obtained from fresh and dried peels. The enzyme SOD acts as an excellent therapeutic agent against diseases caused by ROS and provides essential antioxidant protection against oxidative stress [105]. The results of SOD activities in the present study are comparable to the SOD activities in extracts from fresh mango peels obtained using Tris-HCl buffer as the extraction medium [17]. It was found that the activity of SOD was successfully maintained during drying, as the activities of extracts obtained from dried peels by the same extraction were higher than those of fresh peels. In contrast, opposite results were obtained for PPO activity, where the drying process partially affected the decrease in activity.

Furthermore, activities of various digestive enzymes were found in mango peel extracts. α-Amylase and glucoamylase are starch-degrading enzymes and are among the most frequently used biocatalysts in the food industry [106]. Both enzymes were present in their active forms in all extracts. For both enzymes, air-drying generally resulted in a loss of their activities. Further, proteases are protein-degrading enzymes. Protease activity was detected in most extracts obtained from mango peels using different extraction methods, which coincides with the results in the literature [17,98]. Cellulase activity was generally higher in all aqueous extracts, especially those from fresh peels. Regarding ethanolic extracts, cellulase activities were higher in extracts obtained from dried peels, with the highest activity obtained by the UAE (EtOH) extract from dried peels (124.15 U/g protein). Cellulase plays an important role in the degradation of insoluble cellulose to soluble sugars. It shows great potential for various industries, including agriculture, brewing, textiles, pulp and paper, food, and biofuel production [107]. Most digestive enzyme activities decreased with the drying process of mango peels. However, lipase seems to be resistant to drying, as the activities were higher in the extracts obtained from dried peels regardless of the extraction process and solvent used. These enzymes are also important in various industries, including food, biofuels, detergents, and animal feed [108].

All extracts from mango peels also yielded laccase activity, ranging from 30.78 U/g protein (HAE (H_2_O) extract from dried peels) to 228.88 U/g protein (UAE (H_2_O) extract from fresh peels). Laccases are multifunctional biocatalysts that play an important role in various applications, including the environmental field, the cosmetics industry, food processing, and the textile industry [109]. TGM was also found in its active form in all extracts, although the activity in the HAE (H_2_O) extract from fresh mango peels was significantly higher (120.31 U/g protein) than in other extracts. This can be attributed to the extraction process used (HAE), as the cell wall was successfully disrupted, and thus, the extraction of the TGM in its active form was achieved. In general, TGM activity was lower in extracts from dried mango waste. TGMs are also multifunctional enzymes [110]. From a health perspective, TGMs can reduce allergies, control energy intake from food, and act as mediators in wound healing [111].

The ripening of the mango fruit can lead to a progressive decrease in the activity of antioxidant enzymes, while the activity of digestive enzymes increases [17]. In addition, the extraction procedure, solvent, and operating conditions also significantly impact enzyme activities in the final product [98].

### 3.6. Antimicrobial Activity of Mango Peel Extracts

Infectious diseases represent a growing global problem, making searching for new potential antimicrobial agents increasingly important. Due to the improper and excessive use of synthetic antimicrobials, various pathogenic microorganisms that cause serious diseases have developed resistance [112,113]. For example, *E. coli* can cause different illnesses, including diarrhea, urinary tract infections, respiratory illness, and bloodstream infections [114]. *P. aeruginosa* is associated with causing severe infections and the spread of antimicrobial resistance in vivo [115]. *B. cereus* is known as a foodborne pathogen that can cause two types of gastrointestinal disease: emetic (vomiting) syndrome and diarrheal syndrome [116]. *S. aureus* is responsible for various skin infections up to severe invasive infections of the lungs or heart [117]. *A. brasiliensis* is mostly responsible for allergic reactions and lung infections [118], while *C. albicans* is the main cause of candidiasis and primary fungal infections [119]. Therefore, natural materials and extracts are receiving increasing attention, as they represent a new source of important biologically active compounds with potential antimicrobial properties [120]. For example, extracts from fruit wastes that contain high levels of bioactive compounds with antimicrobial properties, such as mango peels, could be used as effective, alternative, and safe natural remedies [121]. Thus, a comprehensive and comparative antimicrobial study of the different extracts of fresh and dried mango peels was conducted.

#### 3.6.1. Qualitative Antimicrobial Determination of Mango Peel Extracts

The antimicrobial properties of mango peel extracts were preliminarily evaluated using the qualitative disk diffusion method. The susceptibility of Gram-negative (*E. coli*, *P. aeruginosa*) and Gram-positive bacterial species (*S. aureus*, *B. cereus*) and fungi (yeast *C. albicans*, black mold *A. brasiliensis*) to the addition of mango peel extracts was studied. Table 3 shows the measured diameters of the inhibition zone (in mm) of the individual microorganism tested after exposure to the mango peel extracts and ciprofloxacin as a positive control.

*B. cereus* appeared to be the most susceptible to all mango peel extracts among the tested bacterial species. The largest inhibition zone was measured during exposure of *B. cereus* to the EtOH extract obtained by UAE (22 mm) and SF (19 mm) from fresh peels, followed by extracts from dried peels. *B. cereus*, on the other hand, was the most resistant to the UAE (H_2_O) extract from dried peels. The second most sensitive bacterial strain to the mango peel extracts tested was *E. coli*. Exposure of *E. coli* to the UAE (EtOH) extract from dried and fresh peels resulted in the largest zone of inhibition of all the samples tested. The HAE (H_2_O) extract showed the lowest inhibitory effect against *E. coli*. Similar growth inhibition was obtained regarding the antimicrobial efficacy of mango peel extracts on *P. aeruginosa* and *S. aureus* growth. Both bacteria were the least susceptible to the addition of the HAE (H_2_O) extract from fresh and dried peels. Table 3 also shows that ciprofloxacin was effective against all bacterial species tested. The most sensitive strains were *P. aeruginosa* and *B. cereus,* as the highest zone of inhibition was determined.

All the extracts obtained from mango peels successfully inhibited the growth of the tested bacterial species, which is an important observation for the possibility of further use as successful natural antibacterial agents. Generally, higher inhibition zones were achieved with extracts obtained by extraction procedures using EtOH as an extraction solvent or co-solvent. The most outstanding sample with the highest microbial sensitivity when exposed to all four bacterial species was the UAE (EtOH) extract from fresh peels. The extraction solvents were almost completely removed before analysis. Therefore, their influence on the antimicrobial potential of the extracts is negligible. In addition, using the same extraction method, slightly higher growth inhibition was obtained using extracts from fresh mango peels than dried peels.

The results are in agreement with the reviewed literature, which also indicates good antibacterial activity of various extracts from mango peels, with the inhibitory potential of EtOH extracts being higher than those of H_2_O extracts. Methanolic mango peel extract successfully inhibited the growth of *S. aureus*, *Staphylococcus epidermidis*, *P. aeruginosa*, *E. coli*, and *Salmonella* sp. [8,122]. The results of the antibacterial activity of ripe and unripe methanolic mango peel extracts show a larger inhibition zone against all tested bacterial species by ripe mango peel extracts [123]. Also, ethanolic mango peel extract showed inhibition properties against *E. coli*, *S. aureus*, *B. cereus*, *P. aeruginosa*, *S. typhimurium*, and *V. parahaemolyticus* [20,124].

The remarkable antibacterial potential of mango peel extract obtained by various methods is consistent with the fact that various phytochemicals and bioactive compounds are responsible for the disruption of the cell membrane of microbial cells, in addition to their ability to scavenge free radicals, activate endothelial cell migration, and inhibit inflammatory and pain pathways [8]. In contrast, no inhibitory properties of mango peel extracts were found against the tested fungal species (yeast *C. albicans* and black mold *A. brasiliensis*).

#### 3.6.2. Quantitative Antimicrobial Determination of Mango Peel Extracts

Considering the excellent inhibitory effect on the tested bacterial strains (*E. coli*, *P. aeruginosa*, *S. aureus*, and *B. cereus*), the antimicrobial activity was additionally determined after exposure to seven different concentrations of the extracts (0.05–5.0 mg/mL) using a more accurate quantitative microbroth dilution method. MGIR values were determined after 12 and 24 h exposure periods of the bacterial strain to different concentrations of the tested mango peel extracts. In addition, MIC_90_ values were determined based on the MGIR value when at least 90% MGIR was achieved.

**Figure 6:**
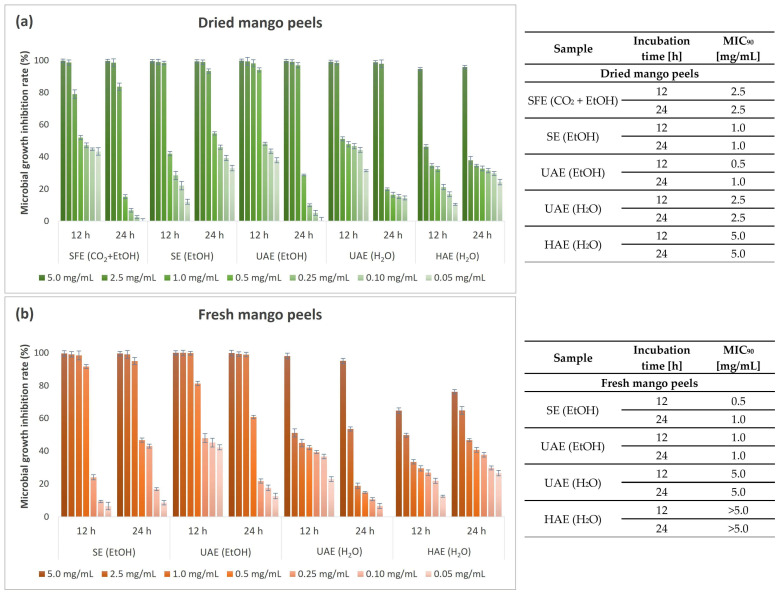
Microbial growth inhibition rate (%) and MIC_90_ values (mg/mL) for different concentrations of mango peel extracts from dried (**a**) and fresh peels (**b**) obtained by different extraction procedures (SFE—supercritical fluid extraction; SE—Soxhlet extraction; UAE—ultrasound-assisted extraction; HAE—homogenization-assisted extraction) with different solvents (CO_2_ + EtOH, EtOH, H_2_O) against Gram-negative bacterium *E. coli*.

Figure 6 shows the results of the antimicrobial study on the growth of Gram-negative bacterium *E. coli* as performed by the microbroth dilution method.

All mango peel extracts, with the exception of the HAE (H_2_O) extract from fresh peels, were very successful in inhibiting the growth of Gram-negative bacterium *E. coli* at the highest concentration tested (5 mg/mL) after 12 h and 24 h of incubation. In general, *E. coli* was more susceptible to ethanolic than aqueous extracts. A slightly higher susceptibility of *E. coli* to aqueous extracts from dried peels was observed, indicating successful retention of bioactive compounds regardless of the drying process. With respect to the HAE (H_2_O) extract from fresh mango peels, the MIC_90_ value of the tested concentrations could not be determined. The analyses would have to be performed at higher extract concentrations to obtain more reliable results. Nevertheless, a relatively high MIGR (76.26%) was achieved at the highest applied extract concentration after 24 h of incubation.

**Figure 7:**
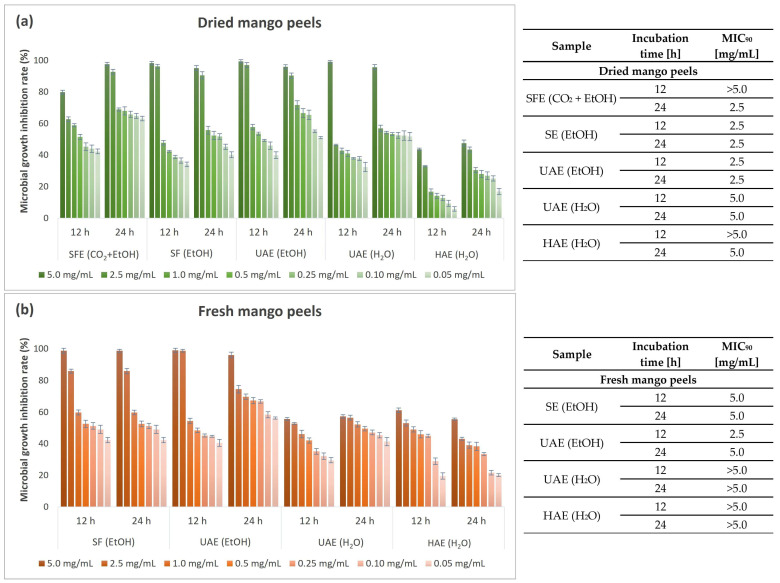
Microbial growth inhibition rate (%) and MIC_90_ values (mg/mL) for different concentrations of mango peel extracts from dried (**a**) and fresh peels (**b**) obtained by different extraction procedures (SFE—supercritical fluid extraction; SE—Soxhlet extraction; UAE—ultrasound-assisted extraction; HAE—homogenization-assisted extraction) with different solvents (CO_2_ + EtOH, EtOH, H_2_O) against Gram-negative bacterium *P. aeruginosa*.

Figure 7 shows the results of the antimicrobial study on the growth of the second Gram-negative bacterial strain, *P. aeruginosa*, as performed by the microbroth dilution method.

*P. aeruginosa* proved to be slightly more resistant to mango peel extracts than *E. coli*. Overall, *P. aeruginosa*, like *E. coli*, was generally more sensitive to ethanolic than aqueous extracts. Among the aqueous extracts, only the UAE (H_2_O) extract from dried mango peels successfully inhibited the growth of the above bacterium, with an effective inhibition of more than 90% at 5.0 mg/mL. Exceptional results were obtained at the lowest concentration (0.05 mg/mL) of the extracts used, with MGIR values above 35% for seven extracts and above 50% for four of the nine tested extracts, with the highest percentage for the SFE (CO_2_ + EtOH) extract (63.11%). This is a remarkable result, because *P. aeruginosa* has become highly resistant to antibiotics. Therefore, new approaches to the treatment of infections are needed. In this regard, mango peel extracts, especially the SFE (CO_2_ + EtOH) extract, would make an important contribution even at lower concentrations.

**Figure 8:**
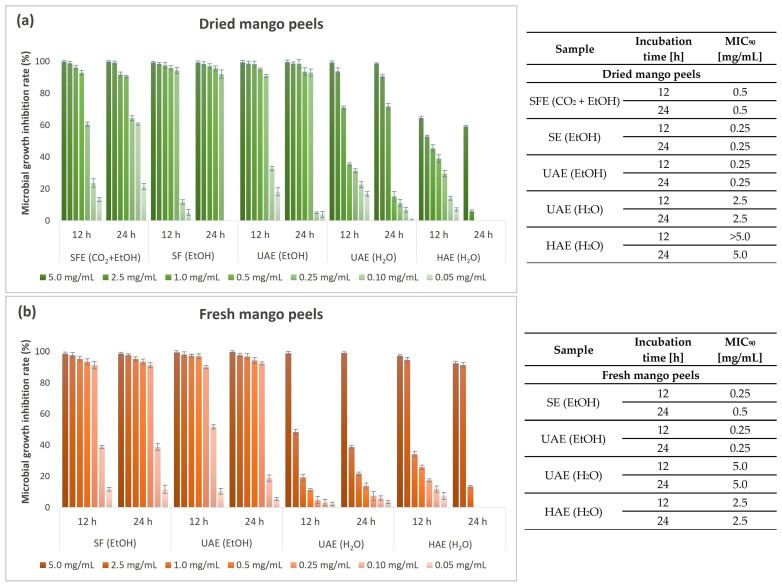
Microbial growth inhibition rate (%) and MIC_90_ values (mg/mL) for different concentrations of mango peel extracts from dried (**a**) and fresh peels (**b**) obtained by different extraction procedures (SFE—supercritical fluid extraction; SE—Soxhlet extraction; UAE—ultrasound-assisted extraction; HAE—homogenization-assisted extraction) with different solvents (CO_2_ + EtOH, EtOH, H_2_O) against Gram-positive bacterium *B. cereus*.

Figure 8 shows the results of the antimicrobial study on the growth of the Gram-positive bacterium *B. cereus* performed by the microbroth dilution method.

Excellent results in terms of inhibitory potential were obtained when Gram-positive bacterium *B. cereus* was exposed to mango peel extracts. Complete inhibition was achieved at the highest concentration tested for all extracts, except the HAE (H_2_O) extract from dried peels. Ethanolic extracts, especially extracts obtained by UAE from fresh and dried mango peels and SE from dried peels, are promising for achieving exceptional growth inhibition of *B. cereus*. Even at a lower added concentration of extracts (0.25 mg/mL), the bacterium was very susceptible to the mentioned extracts, as the MGIR was still above 90%. After 24 h exposure to mango peel extracts, *B. cereus* proved to be highly or completely resistant at lower concentrations of the extracts tested (0.10 and 0.05 mg/mL). Only the SFE (CO_2_ + EtOH) extract still strongly inhibited the growth of the bacterium considered, with 60.75% MGIR at 0.10 mg/mL. *B. cereus* mainly causes intestinal infections but can also cause various eye infections, wounds, skin infections, and respiratory infections. Therefore, mango peel extracts, especially the SFE (CO_2_ + EtOH) extract, would contribute significantly to maintaining effective antimicrobial activity against *B. cereus* growth even at lower concentrations.

**Figure 9:**
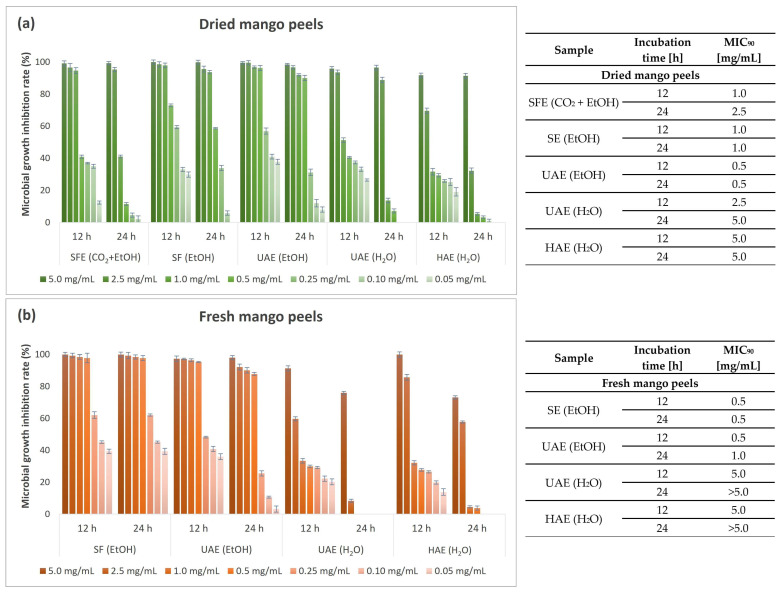
Microbial growth inhibition rate (%) and MIC_90_ value (mg/mL) for different concentrations of mango peel extracts from dried (**a**) and fresh peels (**b**) obtained by different extraction procedures (SFE—supercritical fluid extraction; SE—Soxhlet extraction; UAE—ultrasound-assisted extraction; HAE—homogenization-assisted extraction) with different solvents (CO_2_ + EtOH, EtOH, H_2_O) against Gram-positive bacterium *S. aureus*.

Figure 9 shows the results of the antimicrobial study on the growth of the second Gram-positive bacterium, *S. aureus,* performed by the microbroth dilution method.

The second Gram-positive bacterium tested was *S. aureus*, which was very sensitive to all mango peel extracts after 12 h of incubation. However, after 24 h exposure, the inhibitory potential of the HAE (H_2_O) extract and UAE (H_2_O) extract from fresh peels decreased. Moreover, complete resistance of *S. aureus* was observed at lower added concentrations of the extracts. In contrast, their counterparts, the extracts obtained from dried peels, showed excellent inhibition even after 24 h, although similar complete resistance was obtained at the lowest concentrations. On the other hand, ethanolic extracts were found to possess remarkable antimicrobial properties, with an MGIR above 90% in the case of exposure of *S. aureus* to the UAE (EtOH) extract from dried peels and the SE (EtOH) extract from fresh peels after 24 h of incubation, resulting in 0.5 mg/mL as MIC_90_. In the case of the UAE (EtOH) extract from fresh peels and the SFE (CO_2_ + EtOH) extract, a lower MIC_90_ was obtained after 12 h than after 24 h. This indicates that *S. aureus* can adapt to the bioactive compounds with antimicrobial potential in the extracts during prolonged exposure, to such an extent that the MGIR at 1.0 mg/mL decreased from 94.61 to 41.04% when exposed to the SFE (CO_2_ + EtOH) extract. The antimicrobial properties of plant materials are mainly attributed to the content of various bioactive compounds [125,126,127]. The mechanism of action of PCs is related to bacterial membrane damage, inhibition of virulence factors such as enzymes and toxins, and suppression of bacterial biofilm formation [128]. All phenolic acids investigated in the present study, caffeic acid [129,130], chlorogenic acid [131,132], ellagic acid [133,134], and gallic acid [135,136], were found to have significant antimicrobial activity. In addition, flavonoids have multiple bacteria cellular targets due to their chemical structure. Antibacterial activity is related to their interaction with cell membranes [125]. Flavonoids such as catechin [137,138], hesperidin/neohesperidin [139,140], and rutin [141,142] have antimicrobial potential, according to studies by other authors. Furthermore, mangiferin, an important PC found in all parts of the mango plant, exhibits significant antibacterial activity against both Gram-negative and Gram-positive bacterial strains [19,143].

Thus, we can confirm that the presence of specific PCs (xanthones, phenolic acids, and flavonoids) detected in the extracts obtained contributes significantly to the antimicrobial properties of the samples studied. The SE (EtOH) extracts contained all the PCs tested, with the exception of rutin. Since the content of chlorogenic acid, ellagic acid, caffeic acid, and catechin was very high, it can be confirmed that they synergistically affected the inhibition of the tested bacterial species. The UAE (EtOH) extract of dried peels yielded lower amounts of the tested PCs. However, the synergistic effect of the different PCs was remarkable, as the tested bacterial species were most susceptible to the presence of the UAE (EtOH) extract. On the other hand, the aqueous extracts were rich in certain PCs studied, such as gallic acid, ellagic acid, and catechin. However, chlorogenic acid was only detected in the UAE (H_2_O) extract from the dried peels. Due to the absence of chlorogenic acid in the aqueous extracts, the synergistic effect was not the same as in the case of the UAE (H_2_O) extract from dried peels and ethanolic extracts. Therefore, a lower inhibitory effect on bacterial growth of the aqueous extracts was achieved. In the SFE (CO_2_ + EtOH) extract, all investigated PCs were present except rutin. Since CO_2_ is a non-polar solvent, moderate amounts of PCs were detected, which were successfully extracted by the addition of the polar co-solvent EtOH. Nevertheless, good antimicrobial efficacy was observed. In this case, the antimicrobial properties can most likely be attributed to the synergistic effect of different polar PCs and non-polar compounds.

Although antimicrobial properties are attributed to the whole mango tree due to its content of numerous important bioactive compounds, there are only a small number of studies covering the quantitative antimicrobial activity of mango peel extracts. Zafra Ciprián et al. [144] determined much higher MIC values for the ethanolic extract and the hydroethanolic extract of mango peel, namely over 100 mg/mL for *E. coli* and 25 mg/mL and 12.5 mg/mL, respectively, for *S. aureus*. On the other hand, an MIC of the UAE (EtOH) extract of 0.25 mg/mL was found for *E. coli* [145]. Riberio et al. [146] determined an MIC value of 2.50 mg/mL for *E. coli* and 0.30 mg/mL for *S. aureus* for methanolic mango peel extracts, whereas no inhibitory effect was found for aqueous extracts. Vega-Vega et al. [147] achieved complete inhibition of *E. coli* and *S. aureus* with various extracts of mango peels (methanolic polar, methanolic nonpolar, ethanolic polar, and ethanolic nonpolar) at a concentration of 25 mg/mL. In a study by Espinosa-Espinosa et al. [8], the MIC values for methanolic mango peel extract were 4.0 mg/mL for *E. coli*, *S. aureus*, and *P. aeruginosa*. Rakholiya et al. [148] determined MIC values of 1.25 mg/mL for *B. cereus*, *E. coli*, *P. aeruginosa*, and *C. albicans* with aqueous mango peel extract. Furthermore, mango peel extracts also show improvement in the antimicrobial activity of antibiotics. The addition of ethanolic mango peel extract to three different antibiotics (norfloxacin, erythromycin, and tetracycline) showed a synergistic interaction that resulted in a reduction in *S. aureus* antibiotic resistance [149].

According to the reviewed literature, the quantitative determination of the microbial growth inhibition rate of mango peels has not yet been performed, especially concerning the comparison of extracts from fresh and dried peels and the preservation of antimicrobial potential during the drying of raw peels.

### 3.7. The Most Promising Mango Peel Extracts for Various Applications

The study revealed that most of the bioactive compounds studied were successfully preserved, indicating that the air-drying method is a successful and economical method for the pretreatment of mango waste before the extraction process. Overall, extraction methods using EtOH as a solvent (SE, UAE) or a co-solvent (SEF) (Table 4) were found to be more effective compared with methods using H_2_O (UAE, HAE). In addition, extracts from dried peels generally exhibited a higher profile of bioactive compounds than extracts from fresh mango peels due to the high moisture content in fresh peels (77.74%); the SE (EtOH) extract resulted in the highest TPC content and total content of analyzed PCs. The SFE (CO_2_ + EtOH) extract was the most abundant in PAC content. Furthermore, the UAE (EtOH) extract exhibited the highest antioxidant activity and TP content. Among the analyzed enzymes, antioxidant enzymes SOD and PPO activities stood out. Furthermore, the extracts were also found to contain lipase in a highly active form.

Regarding antimicrobial potential, mango peel extracts, especially ethanolic extracts, represent a potential natural antimicrobial agent as an alternative to conventional antimicrobial agents, as successful growth inhibition was observed in all bacterial species tested. The most susceptible bacterial strain tested was the Gram-positive bacterium *B. cereus*, while the most resistant was the Gram-negative bacterium *P. aeruginosa*.

Besides UAE (EtOH) and SE (EtOH) extracts, the SFE (CO_2_ + EtOH) extract was a promising extract despite the slightly lower inhibition rates for bacterial growth. SFE is considered a green, sustainable, and environmentally friendly process, in which only small amounts of EtOH can be used as a co-solvent. The use of an unconventional extraction technique is becoming increasingly important, as concerns about the impact of organic solvents on the environment and on extracts have increased in recent years.

The UAE (EtOH), SE (EtOH), and SFE (CO_2_ + EtOH) extracts are important for providing antibacterial and antioxidant activity and, in addition, represent a good source of certain enzymes in their highly active form, for potential use in various areas of bioengineering.

## 4. Conclusions

With increasing health and healthy diet awareness, more fruits are being processed, resulting in the generation of enormous amounts of agro-industrial waste from fruit by-products, which has a negative impact on the environment. As they have proven to be a rich source of important bioactive substances with health-promoting benefits, mango peels have been analyzed as a promising source of various bioactive compounds with antioxidant, enzymatic, and antimicrobial activities. Furthermore, a comparison was conducted between extracts from fresh and dried peels to show the effects of an undemanding and inexpensive air-drying method on the maintenance of biological activity. Various conventional (SE, UAE, HAE) and unconventional (SFE) methods were used for the extraction of mango peels. Differences in the extraction yields obtained, bioactive compound content, and biological activity were found due to the different methods, extraction solvents (EtOH, H_2_O, CO_2_ with EtOH as co-solvent), operating conditions, and starting material (fresh or dried peels). Of all the extracts tested, the UAE (EtOH), SE (EtOH), and SFE (CO_2_ + EtOH) extracts from dried mango peels generally stood out as a good source of bioactive compounds in high concentrations. They, therefore, showed good antibacterial, antioxidant, and enzymatic activity.

To the best of our knowledge, there is no comprehensive and detailed study in the literature comparing different extracts of fresh and dried mango peels and their biological activity as obtained by conventional and unconventional methods. Therefore, the present study makes an important contribution to this field. Mango peels thus represent remarkable waste products of the fruit industry that could contribute to the sustainable development of exceptional high-value-added products for various industries, such as pharmaceuticals, cosmetics, and food. For example, bioactive compounds from mango peels are beneficial for the stabilization of cosmetic products due to their antimicrobial and antioxidant activity. They can also provide a soothing effect on the skin. In this case, however, further studies need to be carried out, particularly with regard to the cytotoxicity of the mango peel extracts obtained. Additionally, mango peel extracts can also be used in food packaging to prevent microbial contamination and in the manufacture of food supplements.

## Figures and Tables

**Table 1:** The presence of important phenolic compounds (PCs) in mango peel extracts from fresh and dried peels obtained by different extraction procedures with different solvents (CO_2_ + EtOH, EtOH, H_2_O). SFE—supercritical fluid extraction; SE—Soxhlet extraction; UAE—ultrasound-assisted extraction; HAE—homogenization-assisted extraction. Different letters in the same row indicate significant difference (*p* < 0.05).

Phenolic Compound	Content (µg PC/g DW)						Content (µg PC/g FW)			
	SFE (CO_2_ + EtOH)	SE (EtOH)	UAE (EtOH)	UAE (H_2_O)	HAE (H_2_O)		SE (EtOH)	UAE (EtOH)	UAE (H_2_O)	HAE (H_2_O)
Xanthones										
Mangiferin	0.15 ± 0.01 ^a^	1.73 ± 0.03 ^c^	0.24 ± 0.01 ^a^	39.74 ± 0.54 ^d^	60.43 ± 0.72 ^b^		3.86 ± 0.06 ^c^	24.87 ± 0.41 ^d^	-	1.36 ± 0.02 ^c^
Phenolic acids										
Caffeic acid	2.80 ± 0.12 ^c^	6.60 ± 0.24 ^a^	2.86 ± 0.01 ^c^	5.11 ± 0.12 ^a^	1.81 ± 0.03 ^c^		0.56 ± 0.01 ^d^	0.25 ± 0.01 ^d^	-	0.08 ± 0.00 ^b^
Chlorogenic acid	2.54 ± 0.06 ^a^	26.54 ± 0.17 ^c^	4.69 ± 0.04 ^a^	10.82 ± 0.09 ^b^	-		3.06 ± 0.02 ^a^	3.04 ± 0.04 ^a^	-	-
Ellagic acid	37.00 ± 0.17 ^a^	310.00 ± 1.32 ^c^	96.94 ± 0.65 ^d^	97.23 ± 1.28 ^d^	50.37 ± 0.64 ^e^		531.98 ± 4.09 ^b^	268.89 ± 1.41^c^	539.42 ± 3.50 ^b^	324.49 ± 3.02 ^c^
Gallic acid	58.24 ± 1.23 ^c^	373.53 ± 0.42 ^a^	71.31 ± 0.53 ^d^	405.72 ± 0.75 ^a^	370.12 ± 2.72 ^a^		92.75 ± 0.51 ^d^	37.59 ± 0.55 ^c^	8.03 ± 0.12 ^b^	10.68 ± 0.04 ^b^
Flavonoids										
Catechin	15.35 ± 0.73 ^c^	109.39 ± 0.6 ^a^	67.59 ± 0.27 ^d^	100.13 ± 0.44 ^a^	46.59 ± 0.16 ^d^		31.91 ± 0.12 ^d^	1.95 ± 0.02 ^e^	-	0.38 ± 0.01 ^b^
Hesperidin/Neohesperidin	0.23 ± 0.01 ^a^	2.12 ± 0.04 ^b^	0.59 ± 0.01 ^c^	0.88 ± 0.02 ^c^	2.38 ± 0.03 ^b^		1.45 ± 0.02 ^d^	2.67 ± 0.01 ^b^	0.62 ± 0.02 ^c^	0.69 ± 0.02 ^c^
Rutin	-	-	-	0.80 ± 0.03	-		-	-	-	-
Total content of analyzed phenolic acids	101.08 ± 1.58	716.67 ± 2.14	175.80 ± 1.23	518.89 ± 2.24	422.30 ± 3.38		628.35 ± 4.63	309.77 ± 2.00	547.45 ± 3.62	335.24 ± 3.07
Total content of analyzed flavonoids	15.57 ± 0.74	111.51 ± 0.40	68.18 ± 0.28	101.81 ± 0.49	48.98 ± 0.19		33.37 ± 0.15	4.62 ± 0.03	0.62 ± 0.02	1.07 ± 0.03
Total content of analyzed phenolic compounds	116.80 ± 2.32	829.92 ± 2.57	244.22 ± 1.52	660.43 ± 3.24	531.71 ± 4.29		665.58 ± 4.84	339.26 ± 2.44	548.07 ± 3.36	337.67 ± 3.12

**Table 2:** Activities of certain enzymes in mango peel extracts from fresh and dried peels obtained by different extraction procedures with different solvents (CO_2_ + EtOH, EtOH, H_2_O), expressed as U per g of protein obtained in each extract. SFE—supercritical fluid extraction; SE—Soxhlet extraction; UAE—ultrasound-assisted extraction; HAE—homogenization-assisted extraction. Different letters in the same column indicate significant difference (*p* < 0.05).

	Activity (U/g _protein_ ± SD)									
Sample	α-Amylase	Cellulase	Glucoamylase	Laccase	Lipase	Peroxidase	PPO	Protease	SOD	TGM
Dried mango peels										
SFE (CO_2_ + EtOH)	272.78 ± 7.91 ^d^	37.93 ± 2.69 ^a^	303.04 ± 15.12 ^a^	154.23 ± 9.65 ^b^	2155.15 ± 34.84 ^d^	0.17 ± 0.00 ^a^	329,109.98 ± 370.77 ^d^	5.43 ± 0.08 ^a^	12,400.67 ± 185.61 ^d^	1.05 ± 0.00 ^a^
SE (EtOH)	66.92 ± 4.85 ^c^	72.63 ± 4.23 ^a^	206.00 ± 16.3 ^a^	36.08 ± 1.74 ^a^	5502.79 ± 102.31 ^b^	0.01 ± 0.00 ^a^	99,992.16 ± 143.32 ^c^	6.19 ± 0.31 ^a^	9368.46 ± 42.63 ^b^	3.88 ± 0.02 ^d^
UAE (EtOH)	429.08 ± 12.88 ^e^	124.15 ± 9.63^d^	260.77 ± 6.33 ^a^	173.23 ± 5.32 ^b^	1505.90 ± 34.65 ^a^	0.19 ± 0.01 ^a^	14,036.01 ± 125.32 ^a^	3.08 ± 0.51^b^	106,502.73 ± 421.05 ^a^	1.85 ± 0.01 ^a^
UAE (H_2_O)	110.36 ± 5.64 ^d^	132.86 ± 9.61^d^	232.99 ± 8.51 ^a^	35.05 ± 0.95 ^a^	2885.41 ± 38.21 ^d^	0.06 ± 0.00 ^a^	122,938.17 ± 531.61 ^c^	2.42 ± 0.09 ^b^	17,302.85 ± 105.92 ^d^	1.27 ± 0.04 ^a^
HAE (H_2_O)	9.94 ± 0.52 ^a^	122.51 ± 10.32 ^d^	592.60 ± 22.16 ^b^	30.78 ± 0.75 ^a^	18,643.62 ± 86.96 ^c^	-	407,622.96 ± 540.49 ^b^	-	47,844.09 ± 103.61 ^c^	9.76 ± 0.02 ^b^
Fresh mango peels										
SE (EtOH)	1372.05 ± 53.65 ^b^	22.97 ± 3.51 ^a^	1375.13 ± 81.19 ^c^	41.41 ± 2.36 ^a^	3551.50 ± 58.45 ^e^	0.01 ± 0.00 ^a^	106,299.10 ± 654.61 ^c^	8.05 ± 0.51 ^c^	31,349.82 ± 205.15 ^c^	10.06 ± 0.08 ^b^
UAE (EtOH)	226.39 ± 9.71 ^d^	38.98 ± 2.15 ^a^	584.46 ± 27.77 ^b^	124.74 ± 5.54 ^b^	1160.53 ± 44.99 ^a^	0.14 ± 0.01 ^a^	27,350.05 ± 259.28 ^a^	2.56 ± 0.25 ^b^	95,360.96 ± 329.58 ^a^	1.86 ± 0.03 ^a^
UAE (H_2_O)	414.98 ± 25.91 e	238.14 ± 12.19 ^b^	606.13 ± 32.84 ^b^	228.88 ± 8.13 ^c^	1847.06 ± 21.62 ^a^	0.91 ± 0.05 ^b^	342,415.39 ± 456.32 ^d^	1.69 ± 0.23 ^b^	7445.10 ± 89.51 ^b^	2.99 ± 0.03 ^d^
HAE (H_2_O)	230.26 ± 21.71 ^d^	460.42 ± 18.21 ^c^	665.78 ± 25.48 ^b^	53.24 ± 0.58 ^a^	5189.92 ± 44.21 ^b^	-	430,837.99 ± 653.94 ^b^	-	30,210.78 ± 213.62 ^c^	120.31 ± 0.21 ^c^

**Table 3:** Antimicrobial activity of mango peel extracts from fresh and dried peels obtained by different extraction procedures with different solvents (CO_2_ + EtOH, EtOH, H_2_O) and ciprofloxacin as a positive control, expressed as a zone of inhibition (mm). SFE—supercritical fluid extraction; SE—Soxhlet extraction; UAE—ultrasound-assisted extraction; HAE—homogenization-assisted extraction.

Sample	Zone of Inhibition (mm ± SD)			
	Gram-Positive Bacteria		Gram-Negative Bacteria	
	*E. coli*	*P. aeruginosa*	*B. cereus*	*S. aureus*
Dried mango peel				
SFE (CO_2_ + EtOH)	17 ± 1	10 ± 2	14 ± 1	10 ± 2
SE (EtOH)	18 ± 1	14 ± 1	18 ± 0	15 ± 1
UAE (EtOH)	19 ± 1	14 ± 2	16 ± 2	14 ± 1
UAE (H_2_O)	14 ± 2	7 ± 1	10 ± 1	9 ± 1
HAE (H_2_O)	12 ± 1	9 ± 1	12 ± 1	7 ± 1
Fresh mango peel				
SE (EtOH)	18 ± 2	16 ± 2	19 ± 1	16 ± 1
UAE (EtOH)	21 ± 1	16 ± 1	22 ± 2	15 ± 2
UAE (H_2_O)	17 ± 2	10 ± 1	15 ± 1	11 ± 1
HAE (H_2_O)	10 ± 1	8 ± 1	13 ± 1	7 ± 0
Ciprofloxacin	43 ± 1	55 ± 0	52 ± 1	41 ± 1

**Table 4:** The properties and content of the three most promising mango peel extracts suitable for various applications.

			SFE (CO_2_ + EtOH)	SE (EtOH)	UAE (EtOH)
Total phenolic content (mg GAE/g DW)			1.7 ± 0.0	25.0 ± 0.4	7.9 ± 0.2
Proanthocyanidin content (mg PAC/g DW)			0.4 ± 0.1	0.3 ± 0.1	0.2 ± 0.0
Phenolic compounds present in high concentrations			Chlorogenic acid, ellagic acid, gallic acid, catechin		
Antioxidant activity (% inhibition)			41.4 ± 1.3	59.9 ± 1.8	82.4 ± 1.8
Antibacterial potential (MIC_90_ after 24 h)	Gram-negative bacteria	*E. coli*	2.5	1.0	1.0
		*P. aeruginosa*	2.5	2.5	2.5
	Gram-positive bacteria	*B. cereus*	0.5	0.25	0.25
		*S. aureus*	2.5	1.0	0.5
Total protein content (mg protein/g DW)			0.41 ± 0.04	2.68 ± 0.06	2.95 ± 0.10
Enzymes present in their highly active form			α-Amylase, cellulase, glucoamylase, laccase, lipase, PPO, SOD		

## Data Availability

Data is contained within the article.
